# Modified Tunnel Procedure to Facilitate Ridge Reconstruction of an Extraction Socket Associated with Buccal Dehiscence and Gingival Recession: A Case Report with a 6-year Follow-Up

**DOI:** 10.1155/2023/6614653

**Published:** 2023-05-03

**Authors:** Felice Ascenzi, Andrell Hosein, Emily Ming-Chieh Lu

**Affiliations:** ^1^Centre for Clinical Education, Faculty of Dentistry, Oral and Craniofacial Sciences, King's College London, Guy's Hospital, Great Maze Pond, London SE1 9RT, UK; ^2^Department of Clinical and Molecular Sciences, Università Politecnica delle Marche, Piazza Roma, 22, Ancona 60121, Italy; ^3^Periodontology Unit, Centre for Host-Microbiome Interactions, Faculty of Dentistry, Oral & Craniofacial Sciences, King's College London, Guy's Hospital, Great Maze Pond, London SE1 9RT, UK

## Abstract

**Introduction:**

Extraction sockets associated with buccal dehiscences and gingival recessions pose particular surgical and restorative challenges. In these cases, unassisted healing following flapless tooth extraction results in severe bone and soft tissue deformities and an aesthetic compromise. Root coverage procedures prior to ridge reconstruction may enable predictable alveolar augmentation. *Case Presentation*. This is the first case report describing the utilisation of modified tunnel procedure to facilitate ridge reconstruction consisting of ovate pontic and xenograft, of tooth #25 in a 38-year-old-male. The 6 months and 1-year reviews showed optimal soft tissue aesthetics, 100% root coverage of the tooth #25, and bone augmentation, which enabled placement of 10.0 mm × 4.0 mm (3i) implant in a prosthetically driven position. The 6-year review continued to show favourable clinical outcomes.

**Conclusion:**

Compromised extraction sockets containing buccal dehiscence and associated with gingival recessions may benefit from soft tissue augmentation procedures to enhance the clinical outcome of ridge reconstruction.

## 1. Introduction

Alveolar ridge reconstruction (ARR) provides an effective way to augment extraction sites with bony dehiscences, avoiding complex procedures for implant site development, and thus reducing patient morbidity while ensuring optimal aesthetic outcome.

Recently proposed ridge reconstruction technique consisted of nonabsorbable membrane covering allograft [1]. The presence of the barrier membrane serves to contain the bone graft, as well as space maintenance to prevent unwanted apical migration of the epithelium into the wound during the healing phase, and containment of the bone graft [2]. However, barrier membrane exposure is not uncommon, and contamination may leads to wound healing complications [3].

Extraction sockets associated with buccal dehiscences, as well as gingival recessions pose particular surgical and restorative challenges. Traditional treatment concepts suggest the importance of preserving the buccal bone in order to achieve the desired clinical outcome and, therefore, procedures, such as the “ice-cream cone technique” [4], “socket shield technique” [5], and implementation of SocketKAP and SocketKage [6] devices, have been proposed with the aim of preserving the buccal plate. The “ice-cream cone technique” describes a procedure where collagen membrane has been shaped in a specific way for coverage of the bone graft. However, a key challenge is the prevention of membrane exposure and its resultant complications [4]. The “socket shield technique” suggests retaining the buccal aspect of the root to preserve buccal tissues [5]. However, there is a lack of evidence as to whether this technique would provide a favourable longterm outcome [5]. Finally, the SocketKAP and the SocketKAGE [6] are respective non-resorbable and resorbable devices recently proposed to support sockets with buccal dehiscence, but there is a lack of longterm data to support the benefits of this technique. To date, there is no consensus regarding the specific treatment strategy for the management of extraction sockets associated with a buccal dehiscence [7].

Here, we report on a clinical case where a soft tissue augmentation procedure was performed prior to ridge reconstruction, consisting of immediate ovate pontic and xenograft. There is currently no data on the use of soft tissue augmentation procedures prior to ARR. We challenge the traditional treatment concepts by suggesting that in the presence of augmented soft tissues, the lack of buccal bone provides an ideal environment for maximum bone infill, and thus enable the desired clinical outcomes to be achieved following ridge reconstruction.

### 1.1. Clinical Presentation

**Figure 1:**
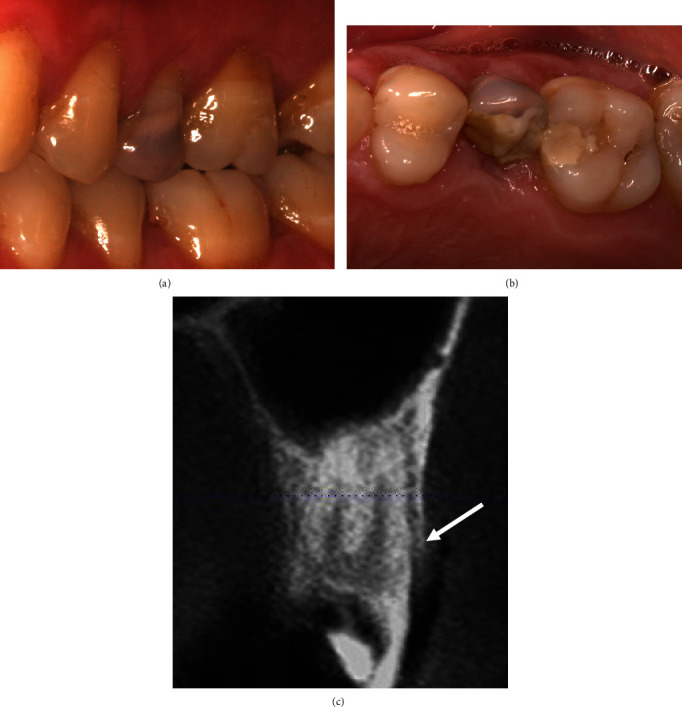
(a) The buccal and (b) occlusal views showed the maxillary left second premolar (tooth #25) was heavily filled and unrestorable, and therefore, indicated for extraction. (c) Baseline cross-sectional CBCT scan showed buccal dehiscence associated with the tooth #25 (the buccal alveolar crest is marked by arrow).

A 38-year-old male patient presented with a heavily restored maxillary left second premolar, tooth #25 (Figures 1(a) and 1(b)), which was associated with a crown fracture and, therefore, deemed unrestorable. The coronal restoration eventually fractured away at the gingival margin, revealing gross caries. Tooth #25 was associated with a mid-buccal recession of 3 mm and probing depth of 3 mm. Clinical assessment revealed gingival recession of the teeth #24, #25, and #26, with a thin tissue phenotype and keratinised tissue width (KTW) ranging 2–3 mm. The baseline clinical outcomes are summarised in Table 1. The baseline dental cone beam computed tomography (CBCT) revealed a buccal dehiscence associated with the tooth #25 (Figure 1(c)).

### 1.2. Case Management

**Figure 2:**
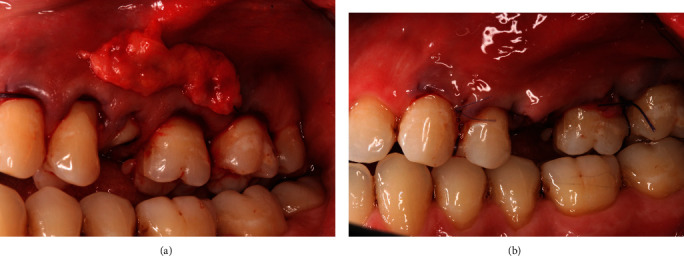
Modified tunnelling procedure extending from the #23–#27 with connective tissue graft for #24, #25, #26 (a). The mucogingival complex was stabilised 1 mm coronal to the CEJ with sling sutures (b).

To address the gingival recession, the modified tunnel procedure [8] was performed. Under local anaesthesia, a continuous split thickness tunnel was made by undermining the buccal mucosa from mesial of tooth #13 to distal of tooth #17 using a tunnelling knife (Hu-Friedy). Care was taken to ensure the papillary tissues were detached, and apical tissues were released appropriately to enable coronal advancement of the soft tissue complex. The palate was anaesthetised, and connective tissue graft (CTG) was harvested via the one incision technique [9]. The CTG was adjusted to ensure even thickness of 0.8–1.0 mm and carefully pulled into the tunnel by way of positioning sutures and stabilised 1 mm coronal to the cemento-enamel junction (CEJ) of teeth #24, #25, and #26 by sling sutures (7/0 Ethilon; Figure 2).

**Figure 3:**
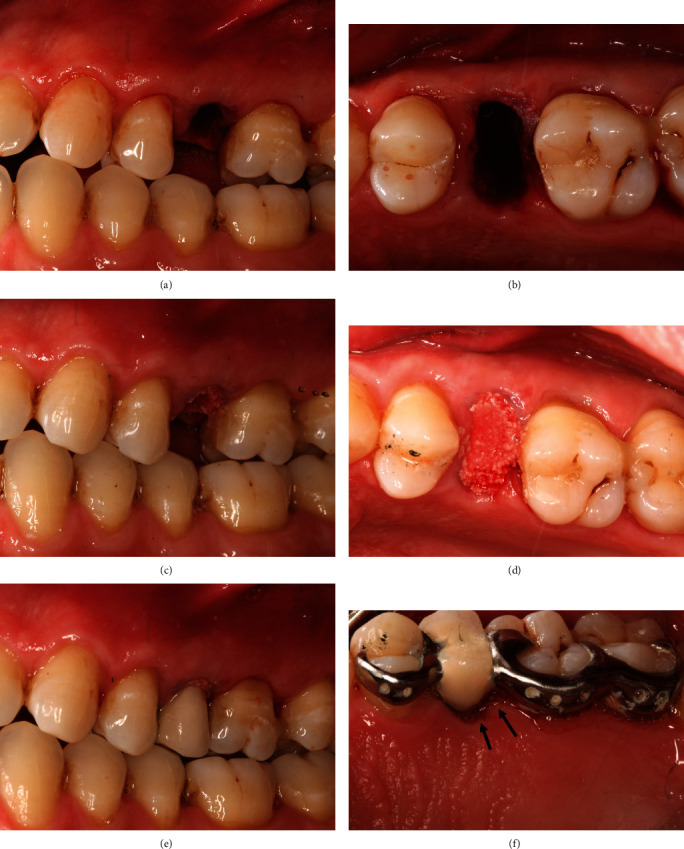
(a and b) Tooth #25 was minimally traumatically extracted. (c and d) The extraction socket was filled with deproteinised bovine bone (Bio-Oss granules, Geistlich). (e and f) The socket sealed using ovate pontic of the Maryland bridge. The pontic has been adjusted to remove all contacts in static and dynamic occlusion. There was a small gap between the pontic and palatal soft tissues (arrowed in f), resulting in escaping of some Bio-Oss particles and, therefore, preventing socket seal in this place.

**Figure 4:**
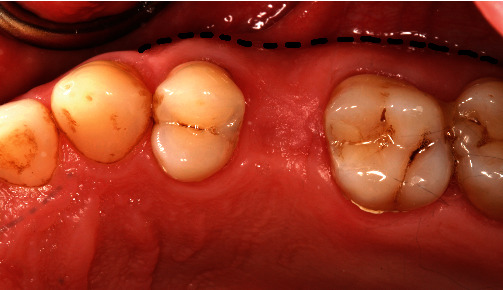
Six months following ridge reconstruction procedure. The buccal dashed line delineates the buccal extention of the ridge.

**Figure 5:**
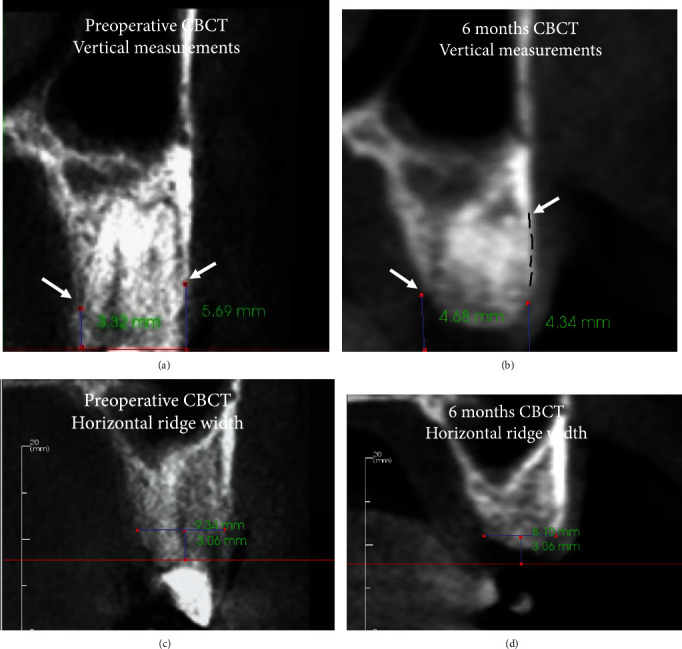
(a and c) Preoperative CBCT and (b and d) 6 months CBCT views were used for linear and horizontal measurements. Six months cross-sectional CBCT showed buccal augmentation and complete infill (delineated in dashed line) to the crest of the buccal defect (b, arrowed). Vertical measurements were taken from the CEJ to the buccal, or lingual alveolar crest (arrowed). (a and b) The preoperative BRH was 5.69 mm, and the postoperative BRH was 4.34 mm. (a and b) The preoperative LRH was 3.32 mm and postoperatively this measured 4.34 mm. (c and d) The preoperative HRW was 9.34 mm and reduced slightly to 8.10 mm postoperatively.

**Figure 6:**
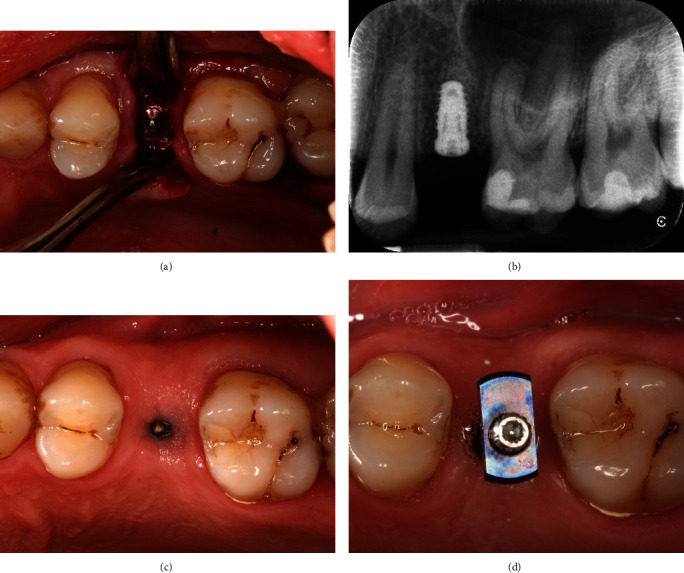
(a) A 10 mm × 4 mm (3i) implant fixture placed. (b) Periapical radiograph showing implant fixture and cover screw in place. (c) Healing at 3 months postoperative showed a soft tissue opening, possibly due to excessive pressive from the pontic of the Maryland bridge, which has been removed. The soft tissue opening allowed the impression coping to be directly connected to the fixture without surgical intervention for implant level impression. (d) The position of the impression coping also illustrates the position of the fixture.

**Figure 7:**
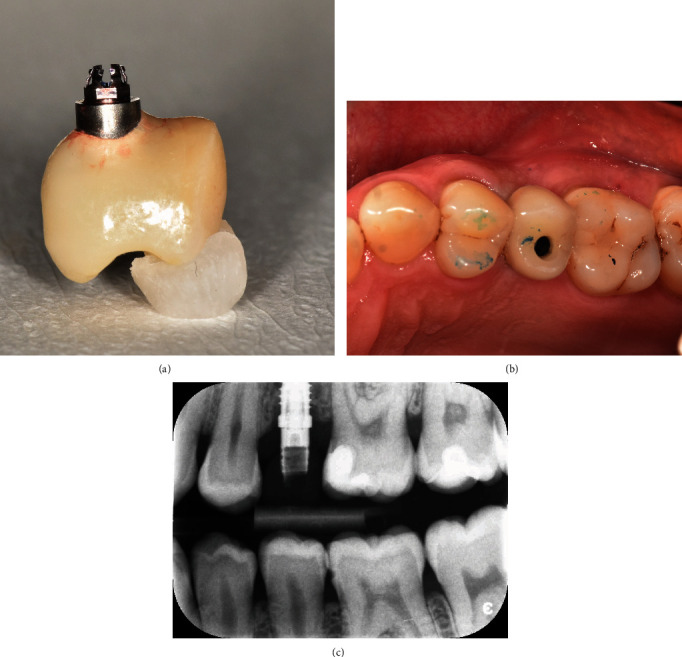
(a) Fabrication of the screw-retained provisional restoration. (b) Occlusal adjustment and installation of the provisional restoration in in situ. (c) Radiograph showed complete seating of the temporary abutment.

**Figure 8:**
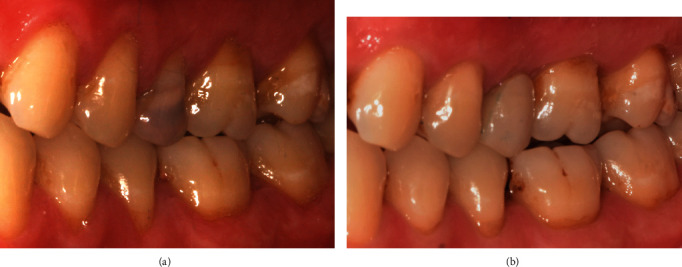
(a) Buccal view of tooth #25 at baseline. (b) Implant provisional restoration in place at 1 year.

At the following appointment, tooth #25 and the adjacent teeth were debrided under local anaesthesia. Minimally traumatic extraction of the tooth #25 was performed (Figures 3(a) and 3(b)). The socket was debrided, irrigated with copious saline, and inspected to confirm the buccal defect. The Maryland bridge was then tried-in, and the ovate pontic was relined with flowable composite. The socket was filled with deproteinised bone substitute (Bio-Oss granules, 0.25–1 mm, Geistlich) up to the level of the free gingival margin (Figures 3(c) and 3(d)), and the Maryland bridge was steam cleaned and cemented (Panavia, Kuraray; Figures 3(e) and 3(f)). The patient was provided with appropriate postoperative instructions. The 6 months review revealed an increase in soft tissue volume, the extent of buccal augmentation, and complete bony infill to the base of the alveolar defect (Figures 4 and 5). The ridge augmentation enabled the placement of 10.0 mm × 4.0 mm (3i) implant, in a prosthetically driven position, which was demonstrated by the angulation of the impression coping (Figure 6) and the emergence profile of the screw-retained provisional restoration (Figure 7). The 1-year review showed optimal soft tissue aesthetics and 100% root coverage of the teeth #24 and #25 and partial root coverage of the tooth #26 (Figure 8).

**Figure 9:**
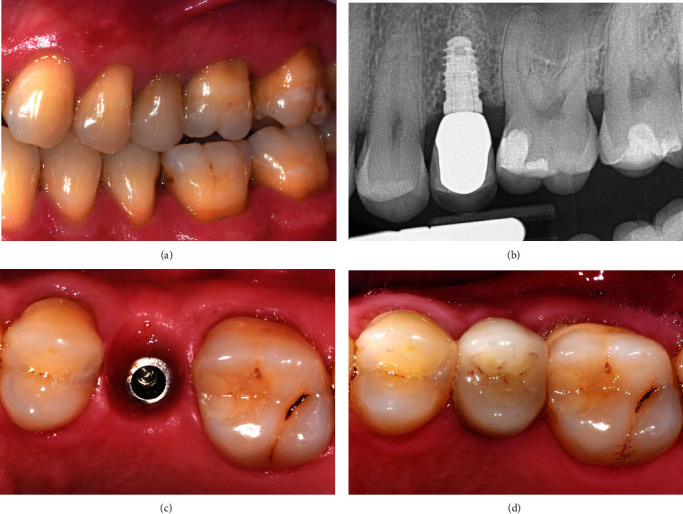
The recent 6-year review. (a) The buccal view showing a good amount of KTW associated with tooth #25 implant. (b) The periapical radiograph showing minimal crestal bone loss. (c and d) Occlusal views highlighting the keratinised tissue thickness around the implant.

Despite a four-year hiatus where the patient lost contact and did not receive maintenance care, the recent 6-year review showed an adequate amount of KTW and thickness associated with tooth #25 implant (Figures 9(a), 9(c), and 9(d)). The periapical radiograph showed minimal crestal bone loss (Figure 9(b)).

## 2. Radiographic Measurements

### 2.1. Linear Assessment

A blinded examiner evaluated the CBCT scans at baseline and 6 months and obtained measurements of horizontal ridge width, mid-buccal, and mid-lingual height using a software package (InVivo v.5.3, Anatomage, San Jose, CA, USA). To ensure accuracy and consistency in measurements, the preoperative and postoperative datasets were registered using the same anatomical landmarks. Vertical measurements were accomplished by using the same global image angulation and reproducible anatomic landmarks on the adjacent teeth, such as the CEJ or crown margins, for maximum consistency between measurements. Horizontal ridge width measurements were made at approximately 3 mm apical to a line connecting the mid-facial zenith of the CEJ of both teeth adjacent to the extraction site (Figures 5(c) and 5(d)). This methodologic decision was driven by clinical relevance since this is often the level at which the restorative platform of a standard bone level implant is placed.

### 2.2. Volumetric Assessment

A blinded examiner performed the volumetric measurements. The preoperative and postoperative CBCT images were superimposed to determine the changes in alveolar bone volume at 6 months. As the first CBCT scan is taken before the extraction, the tooth of interest was “digitally extracted” from the preoperative scan, and the datasets were registered so that the anatomical features are accurately superimposed. DICOM files were processed, and the volumetric measurement were analysed using the Mimics software (Materialise, Leuven, Belgium), by defining a constant volume of interest (VOI) for both preoperative and postoperative datasets. The six boundaries of the VOI were a plane over the crestal bone, a plane over the root apex, a plane over the most external aspect of the buccal and lingual bony plates, and an extension in both the mesial and distal directions of approximately 2–3 mm, for reference purposes to facilitate reliable comparative assessments. The same segmentation settings were used for both the baseline and 6-months DICOM files. The total volume of the VOIs was quantified via subtraction analysis to ascertain the percentage loss of volume that occurred over the 6 months.

## 3. Clinical Outcomes

Ridge reconstruction of the tooth #25 resulted in an increase in KTW by 2 mm and a gain in gingival thickness by 2 mm (Table 1). In addition to the soft tissue augmentation, there was a gain in buccal ridge height (BRH) by 1.35 mm; however, the horizontal ridge width (HRW) and mid-ligual crestal ridge height (LRH) reduced by 1.24 and 1.36 mm, respectively (Table 2). The percentage of the initial alveolar bone volume remaining across zones 0–3, 3–6, and 6–9 mm were 108, 157, and 119%, respectively (Table 2).

## 4. Discussion

Currently, there is no consensus regarding the specific treatment strategy for the management of extraction sockets associated with a buccal dehiscence [7]. This case report demonstrated ridge reconstruction of tooth #25 extraction socket in a patient who presented with deep buccal dehiscence and gingival recession. Modified tunnelling and CTG was performed prior to ARR, which served to (1) provide root coverage of the tooth to be extracted and the adjacent teeth and (2) to maximise soft tissue augmentation and increase KTW at the time of ARR. The recent 6-year review illustrated the favourable longterm clinical outcomes achievable with the proposed technique.

The KTW is the vertical distance from the horizontal line interconnecting two adjacent teeth at the CEJ to the mucogingival junction at the mid-buccal point [10]. The increase in KTW, coupled with the absence of the buccal wall, enabled expansion of the soft tissues, allowing maximum bone augmentation to be achieved, and thus reducing the need for future surgical procedures [11]. While we anticipated 100% root coverage for RT1 defects [12] affecting teeth #24 and #25, the partial root coverage on tooth #26 was due interproximal attachment loss and an RT2 defect [11] at baseline. The resultant soft tissue augmentation at 1 year is also reflected by the increase in KTW and gingival thickness (Figure 8; Table 1).

The ridge reconstruction technique consisted of deproteinised bone graft and an immediate ovate pontic. The bone graft provides a scaffold for bony infill as well as physical support for the soft tissue flap. The ovate pontic stabilises and protects the fibrin clot [13, 14], whereas also enhancing soft tissue augmentation by providing physical support for the soft tissues. The latter is achieved by epithelial attachment to a highly smooth, highly polishable surface of the pontic, which has been relined with flowable composite and steam cleaned. The resultant circumferential socket seal not only maximises bone augmentation but also provides a means by which the pontic protects the bone graft from contamination. The literature suggests that socket grafting alone prevented horizontal width reduction by 2.0 mm; vertical mid-buccal reduction by 1.70 mm and mid-lingual reduction by 1.20 mm, 3–6 months following the intervention [15]. By contrast, socket seal by placement of ovate pontic alone resulted in mean horizontal ridge width dimensional change of 0.90 mm and mean vertical crestal height change of 1.60 mm and at 3 months, suggesting preservation of tissue contour [16]. To date, there is no data on the use of modified tunnel and CTG procedure preceding combined use of ovate pontic and xenograft for ARR.

Ridge reconstruction of the tooth #25 resulted in a mid-buccal crestal bone increase of 1.35 mm. There was also a gain in bone volume across zones 0–3, 3–6, and 6–9 mm, with 108, 157, and 119% alveolar bone remaining, respectively (Table 2). By contrast, comparable compromised extraction sockets containing buccal dehiscences treated with ridge reconstruction comprising of SocketKAP and SocketKAGE devices showed 76% of the initial alveolar bone volume remaining in the crestal 3 mm, whereas zones 3–6 and 6-9 mm both showed 86% of the initial bone volume remaining [6]. This suggests bone volume attenuation, rather than bone augmentation, as demonstrated in our case.

However, there was a reduction in mid-lingual crestal height of 1.4 mm and HRW of 1.2 mm. We speculate that this was due to the slight gap between the edge of the polished pontic and the palatal gingival margin (Figure 3(f), arrowed), allowing some of the Bio-Oss granules to escape as confirmed by the patient. The absence of an adequate socket seal palatally precluded bone augmentation from being maintained [17], and hence the reduction in mid-lingual crestal height and HRW, compared with BRH (Table 2). In other words, the lack of a palatal socket seal resulted in less bone augmentation compared with the buccal aspect. This reinforces the idea that the predictability of the ridge reconstruction technique relies on the presence of an effective circumferential socket seal, which, in this case, is enhanced buccally, by the soft augmentation procedure performed prior to ARR. We challenge traditional treatment concepts, where it has been assumed that the preservation of the buccal bone is paramount to the success of ridge reconstruction procedure [4–6]. We suggest that in the absence of buccal bone, soft tissue augmentation procedures, such as modified tunnel and CTG technique, facilitated maximum bone infill, by enabling expansion of the soft tissues, and thus resulting in the desired outcomes following ARR.

## 5. Conclusion

In this case report, we presented a clinical case showing favourable longterm clinical outcomes following the modified tunnel procedure, which preceded ridge reconstruction of an extraction socket associated buccal dehiscence and gingival recession. The root coverage procedure treated the gingival recession and improved the efficacy of the socket seal by maximising the soft tissue dimension at the time of ridge reconstruction. However, randomised trials are needed to verify these results.

## 6. Summary

**Table:** 

Why is this case new information?	This is the first case describing the use of modified tunnel technique to facilitate ridge reconstruction of a compromised extraction socket
What are the keys to successful management of this case?	Soft tissue management
What are the primary limitations to success in this case?	Incorrect case selection Patient compliance.

## Figures and Tables

**Table 1:** Preoperative clinical outcomes and comparative postoperative values following minimally traumatic extraction of the tooth #16 and ARR.

	Preoperative			Postoperative		
Clinical parameters	Tooth #24	Tooth #25 (indicated for extraction and ARR)	Tooth #26	Tooth #24	Tooth #25 (implant crown)	Tooth #26
Plaque index (%)	0	0	0	0	0	0
Bleeding index (%)	0	0	0	0	0	0
Mid-buccal probing depth (mm)	1	3	1	1	0	2
Mid-buccal gingival recession (mm)	3	3	3	0	0	1
Gingival thickness (mm)	1	0.5	1	2	2.5	2
Keratinised tissue width (mm)	2	2	3	3	4	4
Need for grafting at implant placement	—	Yes	—		No	

**Table 2:** Linear and volumetric radiographic bone measurements for tooth #16.

Linear	Tooth #25		
	Preoperative (mm)	Postoperative (mm)	Difference (mm)
Mid-buccal crestal ridge height	5.69	4.34	1.35
Mid-lingual crestal ridge height	3.32	4.68	−1.36
Horizontal ridge width	9.34	8.10	−1.24

Volumetric	Tooth #25		
	Preoperative (mm^3^)	Postoperative (mm^3^)	% Alveolar bone remaining
0–3 mm	54	59	108
3–6 mm	166	261	157
6–9 mm	261	309	119

## Data Availability

Data supporting this research article is available from the corresponding author or first author on reasonable request.
